# A consensus-based process identifying physical therapy and exercise treatments for patients with degenerative meniscal tears and knee OA: the TeMPO physical therapy interventions and home exercise program

**DOI:** 10.1186/s12891-019-2872-x

**Published:** 2019-11-04

**Authors:** Clare E. Safran-Norton, James K. Sullivan, James J. Irrgang, Hannah M. Kerman, Kim L. Bennell, Gary Calabrese, Leigh Dechaves, Brian Deluca, Alexandra B. Gil, Madhuri Kale, Brittney Luc-Harkey, Faith Selzer, Derek Sople, Peter Tonsoline, Elena Losina, Jeffrey N. Katz

**Affiliations:** 1 Orthopaedic and Arthritis Center for Outcomes Research (OrACORe) and Policy and Innovation eValuation in Orthopedic Treatments (PIVOT), Department of Orthopaedic Surgery, Brigham and Women’s Hospital, 75 Francis Street, BTM 5016, Boston, MA 02115 USA; 2 Department of Rehabilitation Services – Physical and Occupational Therapy, Brigham and Women’s Hospital, Boston, MA USA; 3 Department of Physical Therapy, School of Health and Rehabilitation Sciences, University of Pittsburgh, Pittsburgh, PA USA; 4 Centre for Health, Exercise and Sports Medicine, School of Health Sciences, University of Melbourne, Melbourne, Australia; 5 Department of Rehabilitation and Sports Therapy, Cleveland Clinic, Cleveland, OH USA; 6 UBMD Department of Orthopaedics and Sports Medicine, Jacobs School of Medicine and Biomedical Sciences, State University of New York at Buffalo, Buffalo, NY USA; 7 Harvard Medical School, Boston, MA USA; 8 Department of Biostatistics, Boston University School of Public Health, United States of America, Boston, MA USA; 9 Section of Clinical Sciences, Division of Rheumatology, Immunology and Allergy, Brigham and Women’s Hospital, Boston, MA USA; 10 Departments of Epidemiology and Environmental Health, Harvard T. H. Chan School of Public Health, Boston, MA USA

**Keywords:** Osteoarthritis, Meniscal tear, Randomized trial, Physical therapy, Home exercise program

## Abstract

**Background:**

Knee osteoarthritis (OA) is prevalent and often associated with meniscal tear. Physical therapy (PT) and exercise regimens are often used to treat OA or meniscal tear, but, to date, few programs have been designed specifically for conservative treatment of meniscal tear with concomitant knee OA. Clinical care and research would be enhanced by a standardized, evidence–based, conservative treatment program and the ability to study the effects of the contextual factors associated with interventions for patients with painful, degenerative meniscal tears in the setting of OA. This paper describes the process of developing both a PT intervention and a home exercise program for a randomized controlled clinical trial that will compare the effectiveness of these interventions for patients with knee pain, meniscal tear and concomitant OA.

**Methods:**

This paper describes the process utilized by an interdisciplinary team of physical therapists, physicians, and researchers to develop and refine a standardized in-clinic PT intervention, and a standardized home exercise program to be carried out without PT supervision. The process was guided in part by Medical Research Council guidance on intervention development.

**Results:**

The investigators achieved agreement on an in-clinic PT intervention that included manual therapy, stretching, strengthening, and neuromuscular functional training addressing major impairments in range of motion, musculotendinous length, muscle strength and neuromotor control in the major muscle groups associated with improving knee function. The investigators additionally achieved agreement on a progressive, protocol-based home exercise program (HEP) that addressed the same major muscle groups. The HEP was designed to allow patients to perform and progress the exercises without PT supervision, utilizing minimal equipment and a variety of methods for instruction.

**Discussion:**

This multi-faceted in-clinic PT program and standardized HEP provide templates for in-clinic and home-based care for patients with symptomatic degenerative meniscal tear and concomitant OA. These interventions will be tested as part of the Treatment of Meniscal Tear in Osteoarthritis (TeMPO) Trial.

**Trial registration:**

The TeMPO Trial was first registered at clinicaltrials.gov with registration No. NCT03059004 on February 14, 2017. TeMPO was also approved by the Institutional Review Board at Partners HealthCare/Brigham and Women’s Hospital.

## Background

Symptomatic knee osteoarthritis (OA) and its attendant pain and functional limitations affect approximately 14 million adults in the United States (US) [1, 2]. Meniscal tears are present in 60–90% of persons with symptomatic knee OA [3, 4]. While physical therapy (PT) is a widely accepted first-line treatment for patients with knee OA and meniscal tear [5–7], multiple musculoskeletal rehabilitation treatment regimens exist in the literature [8–16], and a consensus has not been reached on which elements of a PT program or home exercise program provide the best results in this patient population. PT is also widely used and supported in the literature for patients with OA to improve function [17–32] and pain. There is little literature on physical therapy interventions for patients with meniscal tear, or meniscal tear in the setting of knee OA. Therefore, understanding which elements of a PT intervention, or home exercise program are most effective for patients with meniscal tear and concomitant OA, and the role of contextual factors in treatment outcomes, remains an important research gap.

Our research team has launched a clinical trial entitled the Treatment of Meniscal Tears in Osteoarthritis (TeMPO) (ClinicalTrials.gov Registration No. NCT03059004) to evaluate the outcomes of in-clinic physical therapy intervention and self-guided home exercise program for persons with meniscal tear in the setting of OA. The TeMPO trial study protocol has been published previously and meets the SPIRIT (Standard Protocol Items: Recommendations for Interventional Trials) guidelines for reporting clinical trial study protocols [17]. However, given the lack of a consensus on which elements of a PT program should be included for treatment of meniscal tear in the setting of symptomatic knee OA, our group faced the challenge of developing strong evidenced based interventions that would help close this research gap.

This paper reports our process for developing an in-clinic PT intervention and a self-administered home exercise program (HEP) for the TeMPO Trial using a dual approach of best available published evidence and clinician experience in our field of interest. We sought to describe our process in sufficient detail such that it could be replicated and modeled by other groups seeking to develop a rigorous trial intervention in the absence of clear clinical recommendations. Our process aligns with many aspects of the Medical Research Council (MRC) guidance [18]. In particular, we formally evaluated evidence of effectiveness and we used our expert panel’s extensive clinical experience to address feasibility of the intervention and home exercise program. Our process was also informed by that of Bennell et al., in the development of a PT intervention for OA [19].

The two main goals of the project were: 1) to create a PT intervention that utilized best published evidence available and consensus from an expert panel of experienced PTs, physicians and researchers and 2) to create a self-administered progressive HEP that could be performed at home without any direct contact by a physical therapist in a target population of adults with degenerative meniscal tear and concomitant knee OA. For this reason, we will refer to the programs in this manuscript as the TeMPO in-clinic intervention and the TeMPO HEP.

## Main text

### Design overview

The development of the TeMPO in-clinic and HEP programs consisted of six phases. In phase one, a core group of physical therapists, physicians and research methodologists invited a group of physical therapists from four academic US centers to form an expert panel that would guide the creation of both TeMPO programs. This group communicated extensively on conference phone calls and attended in person meetings throughout the trial development.

The second phase involved a review of relevant literature on physical therapy interventions and strengthening programs designed for patients with both degenerative meniscal tear and concomitant knee OA. In the third phase, the research team administered an e-mail survey to a convenience sample of experienced physical therapists in several US states to gain a better understanding of the types of PT treatments used in our target population.

In the fourth phase, the initial expert panel was expanded to add more orthopaedic surgeons, rheumatologists, researchers and physical therapists from the four study sites to form the trial research team. This expanded panel participated in a series of conference calls and two in person meetings to develop, review, and revise the components of the TeMPO program. These calls emphasized both the validity of the intervention elements as well as their feasibility. As many of the panel members had extensive clinical experience as well as trial experience, they were able to ensure that the interventions would be feasible both for the study as well as for subsequent clinical application.

During the fifth phase, the research team and physical therapists created a standardized instructional video and hard copy program booklet to guide patients who would be completing the TeMPO exercises without direct physical therapist supervision. The team also created a Manual of Operations of the physical therapy in-clinic intervention for the TeMPO trial. Phase six included focus group sessions with the research team and potential patients to solicit feedback for final edits to the TeMPO exercise program and its instructional materials.

### Expert panel composition

The initial core group of 10 members included physical therapists, rheumatologists and methodologists from four collaborating US sites including Brigham and Women’s Hospital in Boston, MA, Cleveland Clinic in Cleveland, OH, University of Buffalo Medical Center in Buffalo, NY and University of Pittsburgh in Pittsburgh, PA. One member was from Melbourne, Australia. This core expert panel developed the foundation of the TeMPO interventions. Additional physical therapists, orthopedic surgeons, methodologists, an exercise physiologist and research staff -- numbering about 20 individuals -- were invited to further develop the TeMPO interventions. The full 30-member panel attended the two in person meetings. The clinicians involved in this process had extensive experience with the target population and as a result were able to ensure that the interventions would be feasible for implementation in trials, clinical practice and self-administered home programs.

### Data collection procedures and sources

#### Literature review

Research staff in conjunction with the physical therapists performed a literature review to identify studies that addressed the efficacy of PT in persons with degenerative meniscal tear and concomitant OA. The search words included: “meniscal tear,” “osteoarthritis,” “physical therapy,” “exercise,” and “rehabilitation.” We also searched for studies of manual therapy used in the treatment of knee OA without specific mention of meniscal tear and the use of manual therapy in reported clinical guidelines. Specific search terms included “meniscectomy,” “knee OA,” “joint mobilization,” “manual therapy,” “physical therapy,” “stretching,” and “massage.” We conducted these searches within Google Scholar and PubMEd and also examined professional organization websites.

#### Brief survey of PTs

We administered a survey regarding current PT clinical practice for patients with meniscal tear in the setting of concomitant OA to a convenience sample of experienced physical therapists across the US. The survey clinicians were identified by our expert panel and their professional contacts from regions throughout the US. Respondents provided information on (1) the therapeutic exercises they used for patients with knee OA and meniscal tear; (2) the number of minutes dedicated to therapeutic exercise in a typical PT session with this patient group; (3) the use of manual therapy administered by a physical therapist (such as joint mobilization, manual stretching, or soft tissue mobilization) (4) minutes dedicated to manual therapy when indicated in a typical PT session; and (5) any other interventions typically utilized in this patient population such as thermal agents, cryotherapy or ultrasound (Additional file 1). A copy of the complete survey is included in Additional file 1.

#### Panel process with emails, conference calls and in-person meetings

After we compiled the information from the literature search and the PT survey, we arranged a series of e-mails, surveys, telephone calls, and monthly team-wide conference calls to allow the expert panel to define the goals and outcomes of the project and the timeline for creating, developing and revising the in-clinic PT intervention and the elements of the HEP prior to the final in-person consensus meeting. Over the course of 2 yrs, we held monthly conference calls and two in-person meetings, in which all team members gathered for a day-long conference. Each participant was encouraged to contribute to the discussion and to comment by email on provisional decisions. The second meeting was held to review and reach 100% complete investigator consensus on details of the TeMPO program. We chose this approach over more formal methods of achieving agreement (e.g. Delphi method) because our approach permitted more fluid and interactive discussions than are generally afforded by more formal approaches.

After the final in person meeting, a core group of PTs video-taped the TeMPO Home Exercise Program. Research Assistants and PTs created instructional TeMPO exercise pamphlets for future patients and a manual of operations for physical therapists conducting the in-clinic TeMPO PT intervention. Once the videos, pamphlets, and the manual for operations were completed, they were reviewed by the expert panel and by a focus group of potential patients. Edits and revisions were made accordingly to improve the clarity of the materials. Final materials for the TeMPO home exercise programs were created in various learning mediums including USB flash drive, website, and hard copy pamphlets. The HEP pamphlet and links to the video have been published previously as Additional File I in Sullivan et al., 2018 [17]. The in-clinic PT program’s manual of operations for exercises has also been published previously as Additional File III in Sullivan et al., 2018 [17].

## Conclusions

### Results of literature review

The literature search confirmed that neither the American Academy of Orthopedic Surgeons nor the American Physical Therapy Association (APTA) nor any other organization we could identify has developed clinical practice guidelines specifically for rehabilitative management of degenerative meniscal tear in the setting of knee OA [20, 21]. The current established PT guidelines address non-operative management of knee OA or degenerative meniscal tears [20, 21] without OA. In the absence of clear clinical guidelines for PT-based treatment of meniscal tear in the setting of knee OA, randomized controlled trials (RCTs) comparing the efficacy of PT alone to meniscectomy and PT were identified as the most relevant body of work. The PT interventions in these trials were developed rigorously and shown to reduce pain in this patient population. These trials demonstrated that subjects assigned to PT-based treatment improved in pain scores by ~ 1 standard deviation (Table 1). As a result, these RCTs acted as a starting point for the TeMPO PT regimen [10, 15, 22–30]. We acknowledge that the observed improvements (in PT and surgical arms) include both direct and contextual effects.

**Table 1:** Overview of randomized controlled trials of arthroscopic partial meniscectomy (without or without physical therapy) vs. physical therapy for adults with non-traumatic meniscal tear, published by the start of the TeMPO Trial

Author/ Year	Population	Compar- ison	n	Therapist Directed Therapy	Manual Therapy	Thera- peutic Exercise	Bicycle Warm-up	Progression	Stretching	Additional Home Exercise	Improvement in both groups in primary outcome (mean, SD)
Herrlin 2007 [31]	Non-traumatic MT^; mean age 56 years; age range 45–64 years	APM with PT vs. PT alone	90	Yes	No	Yes	Yes	Yes	No	Yes	*Outcome:* Change in KOOS pain (0–100) at 8 weeks --APM: 33 SD (24.34) * --PT: 24 (SD 27.30) *
Katz 2013 [24]	MT, mild-to-moderate OA; mean age 58.4 years; all participants ≥45 years	APM with PT vs PT alone	351	Yes	Yes	Yes	Yes	Yes	Yes	Yes	*Outcome:* Change WOMAC Fuction (0–100) at 6 months --APM: 20.9 (SD 19.42) ** --PT: 18.5 (SD 19.90) **
Yim 2013 [30]	Degenerative MT; mean age 53.8 years; age range 43–62 years	APM with PT vs. PT alone	102	Yes	No	Yes	Yes	No	Yes	Yes	*Outcome:* Change in VAS Pain scale (0–100) at 2 years --APM: 34 (SD 25.46) *** --PT: 32 SD (21.21) ***
Gauffin 2014 [22]	Patients with knee pain and MT; mean age 54 years; age range 45–64 years	APM with PT vs PT alone	150	Yes	No	Yes	Yes	Yes	No	Yes	*Outcome:* Change in KOOS Pain scale (0–100) at 12 months --APM: 29 (SD 24.49) **** --PT: 20 (SD 26.44) ****
Stensrud 2015 [27]	Unilateral, degenerative MT and no or mild OA; mean age 49 years; age range 35–60 years	APM vs. PT.	82	Yes	No	Yes	Yes	Yes	No	Yes	*Outcome:* Change in isokinetic knee extension peak torque (Nm) at 3 months --APM: 0.5 (SD 26.12) ** --PT: 25.2 (SD 23.88) **
Kise 2016 [25]	Patients with degenerative MT; mean age 49.5 years; age range 35.7–59.9 years	APM vs. PT	140	Yes	No	Yes	Yes	Yes	No	Yes	*Outcome:* Change in KOOS_4_ scale (0–100) at 2 years --APM: 24.4 (SD 15.10) ** --PT: 25.3 (SD 14.86) **

^MT = meniscal tear

^*^ SD values at baseline and 6-month follow-up for APM and PT groups estimated using method proposed by Wan et al. [43]

SD_d_ for the difference in KOOS scores for each treatment was estimated using the following equation: ${SD}_d=\sqrt{\delta_{BL}^2+{\delta}_{6\  months}^2}$

^**^ SD estimated from 95% CI where ${SD}_d=\frac{\left(\sqrt{n}\ast half- width\ of\ 95\% CI\right)}{1.96}$

^***^ Only SD (*δ*_*BL*_) at baseline given, therefore, the SD_*d*_ for the difference in VAS scale (0–100) was estimated by the following equation: ${SD}_d=\sqrt{2{\delta}_{BL}}$

^****^ SD given at baseline and follow-up; SD_d_ for difference in KOOS scores for each treatment estimated using the following equation: ${SD}_d=\sqrt{\delta_{BL}^2+{\delta}_{12\  months}^2}$

The results of the literature search regarding RCTs of PT and meniscal surgery [10, 15, 22, 24, 26–32] pointed to progressive strengthening as the most common element of PT programs directed at meniscal tear and OA. The information collected from the literature search (Table 1) and the PT survey identified the muscles to target and the components of the intervention and home exercise program. The muscle groups targeted by strengthening programs typically included quadriceps, hamstrings, gluteus maximus, and gluteus medius (Table 2). Programs frequently included a bicycle warmup, therapeutic exercise progression, and directions for exercises to be completed at home (Table 1).

**Table 2:** Muscle groups and weight bearing functional exercises targeted by the physical therapy regimens of the randomized controlled trials of arthroscopic partial meniscectomy versus physical therapy in middle age persons

Author/year	Muscles targeted				Weight-bearing Exercises Functional/Neuromuscular
	Glut max	Glut med	Quad	Hamstring	
Gauffin 2014 [22]	Yes	Yes	Yes	Yes	Yes
Herrlin 2007 [31]	Yes	Yes	Yes	Yes	Yes
Katz 2013 [24]	Yes	No	Yes	Yes	Yes
Kise 2016 [32]	Yes	Yes	Yes	Yes	Yes
Stensrud 2015 [27]	Yes	Yes	Yes	Yes	Yes
Yim 2013 [30]	Yes	Yes	Yes	Yes	Yes

Only one of the 6 trials initially reviewed (Table 1) utilized manual therapy as part of the general PT regimen. The expert panel considered a more recent study that suggested that manual therapy coupled with exercise/strengthening based therapy appeared more efficacious than exercise/strengthening therapy alone in patients with OA [33] and expanded the literature search for the manual therapy portion of the intervention to include clinical trials with these types of manual therapy techniques utilized in the setting of knee OA or meniscal tear (Table 3). This updated review of the literature revealed that manual stretching, soft tissue mobilization (massage), and joint mobilization were all widely used in RCTs involving meniscal tear treatment (Table 3).

**Table 3:** Overview of manual therapy procedures in randomized controlled trials of osteoarthritis or meniscal tear and osteoarthritis

Author/year	Soft tissue mobilization	Joint mobilization	Stretching
Katz 2013 [24]	Yes	Yes	Yes
Goodwin 2003 [15]	Yes	Yes	No
Abbott 2015 [44]	Yes	Yes	Yes
Deyle 2005 [45]	Yes	Yes	Yes
Moss 2007 [46]	No	Yes	No
Perlman 2006 [47]	Yes	No	No
Weng 2009 [48]	No	No	Yes

### Results of PT survey

A convenience sample of 21 physical therapists across 8 states responded to our survey regarding typical treatments for patients seeking treatment for degenerative meniscal tear in the setting of OA. The 21 physical therapists had a mean of 20 years of experience (range 6 to 38 years) and practiced in 8 states (CA, CO, DE, MA, NY, PA, TX, UT). Thirteen PTs practiced in academic settings, 7 in private practice, and one in both.

All 21 respondents indicated that they typically include a strengthening program and 19 of 21 regularly incorporate manual therapy as listed above into their treatment strategy. The respondents reported the mean appointment duration as 41 min (standard deviation (SD) 14), the mean duration of strengthening exercises as 24 min (SD 10) and the mean duration of manual therapy as 12 min (SD 4). The results of these findings were incorporated into the TeMPO in-clinic intervention and home exercise program.

### Results of expert panel deliberation

The panel defined the research objective as creating an effective, flexible, and realistic in-clinic PT intervention for patients with meniscal tear and concomitant OA pain and an efficacious, standardized, and practical HEP that could be performed independently and safely at home without direct input from a physical therapist. Based on the findings of the expert panel, literature search and the clinical PT practice survey, the panel established components of the TeMPO in-clinic PT intervention and HEP. The targeted muscles for strengthening in both programs included gluteus maximus, gluteus medius, quadriceps, and hamstring muscle groups. The targeted muscles for stretching included quadriceps femoris, hamstrings, and gastrocnemius.

Neuromuscular/functional weight bearing exercises were also included (Table 4). Exercises were designed to adhere to the strengthening guidelines established by the APTA and American College of Sports Medicine (ACSM) [21, 34]. Additionally, the literature and general practice standards lean toward progressive non-weight-bearing and weight-bearing strengthening, neuromuscular training, and weight-bearing functional exercises including unilateral stance activities to improve knee stability. The TeMPO programs were designed to adhere to these features as well. When considering the functional anatomy of the meniscus as a passive stabilizer, the goal of neuromuscular and unilateral weight-bearing therapeutic exercise is achieving greater dynamic stability to compensate for the deficiency of the meniscus. While strengthening is important to create force and aid function, stabilization is equally important to prevent unwanted movement and thus minimizing shear stress at the knee.

**Table 4:** Protocolized home exercise program and progression

Exercise target*	Initial	Intermediate	Advanced
Stretches	Hamstrings 2x30s** Quadriceps 2x30s	Hamstrings 2x30s Quadriceps 2x30s	Hamstrings 2x30s Quadriceps 2x30s
Gluteus Maximus strengthening	Bent over hip extension with knee bent without weight; OR *Bridging*	Bent over hip extension with knee bent with weight (1–5 lbs)	Bent over hip extension with knee bent with weight (6–10 lbs)
Gluteus Medius strengthening	Side-lying straight leg lift without weight; OR *Clamshell*	Side-lying straight leg lift with weight (1–5 lbs)	Side-lying straight leg lift with weight (6–10 lbs)
Quadriceps strengthening	Straight leg raise without weight; OR *Seated knee extension without weight*	Straight leg raise with weight (1–5 lbs); OR *Seated knee extension with weight (1–5 lbs)*	Straight leg raise with weight (6–10 lbs); OR *Seated knee extension with weight (6–10 lbs)*
Hamstrings strengthening	Standing knee bends without weight	Standing knee bends with weight (1–5 lbs)	Standing knee bends with weight (6–10 lbs)
Functional exercises	Mini wall squats	Regular chair squat	Staggered leg chair squat

^*^All exercises, except where indicated are done in 3 sets of 12 repetitions (reps) 4 times per week. Patients are encouraged to begin at as low as 3 sets of 8 reps and work their way to 12 reps per set as tolerated

^**^ 2x30s refers to two reps of a 30 s hold for each stretch

While the initial literature review identified just one studies of joint and/or soft tissue mobilization in this population, the expert panel considered the more recent studies of mobilization in OA [33], the finding that over 90% of respondents to the PT survey used mobilization in this population and the findings of Table 3. Thus, the panel opted to include joint and soft tissue mobilization in the TeMPO in-clinic PT program.

#### Program progression

The expert panel discussed methods for tracking patients’ progression from one level of resistance and/or repetitions to the next. In the HEP, the panel determined that TeMPO participant materials should describe the perceived level of symptoms and muscle fatigue as appropriate indicators to progress to additional weight. Rather than employing a specific scale (such as the Borg Rating of Perceived Exertion Scale [35]), we instruct participants to progress first by increasing exercise repetitions and sets (from 3 sets of 8 repetitions to 3 sets of 12 repetitions) before adding weight in increments of one pound and returning to 3 sets of 8 repetitions**.** Study participants are instructed to remain at the same level of difficulty until “you can complete 3 sets of 8-12 repetitions without soreness or discomfort.” We also caution study participants not to advance in exercise difficulty if they experience muscle soreness or increased joint pain within 24 h following an exercise session. If TeMPO participants experience pain with any of the HEP, specific guidelines regarding rest, ice and hold on progression are delineated in the pamphlet and videos. Pages vi – ix in the TeMPO Guide to Home Exercises (Additional File I of Sullivan et al. [17]) detail the instructions exactly.

#### Program duration

The panel carefully considered exercise program duration and repetitions/sets. The panel determined that both the HEP and the in-clinic PT intervention should last no longer than 30 min to optimize adherence and conform to the time frame permissible in routine PT practice in the US. This consideration was informed in part by the emphasis on feasibility in the MRC guidance paper [18]. The panel chose to include elements routinely recommended by current PT literature for the in-clinic program and wherever feasible within the HEP including: warm-up, stretching, and progressive strengthening exercises [20]. Of note, the warm up does not count toward the 25–30-min window of time spent with the therapist in the PT in-clinic program. The warm up is optional with the HEP and not required because many participants may not have access to a stationary bicycle at home. The exercises are to be performed with an emphasis on multiple repetitions with gradual increases in weight as tolerated to avoid injury at home with the self-administered program [21].

#### HEP parameters

The panel decided on overall parameters to guide the final form of the HEP: 1) The program should be accessible and safe for persons of varied ages and presenting with a variety of concomitant comorbid conditions; 2) Minimal equipment should be required; 3) Participants should be able to complete the exercises within a fairly short time period, defined as 25 min by the panel; and 4) Participants should be able to progress the program or adjust accordingly through standardized and understandable thresholds.

Recommendations**:**

#### In-clinic PT intervention

The in-clinic PT intervention consists of three primary components: 1) unsupervised bicycle warm up; 2) manual therapy comprising joint mobilization, stretching, and soft tissue mobilization and 3) therapeutic strengthening and functional exercises. The manual therapy portion of the in-clinic PT is utilized by the therapist and includes joint mobilization of patella-femoral, tibio-femoral and tibio-fibular joints; and manual stretching of quadriceps, hamstrings, hip flexors, iliotibial band and gastrocnemius. Soft tissue mobilization includes effleurage, deep friction and petrissage massage of tight muscles. The therapeutic strengthening elements of the intervention recommend use of exercise machines and resistance bands commonly available in PT clinics. Study therapists are also given an extensive list of recommended exercises to choose from in the manual of operations for the TeMPO RCT. The complete list of the recommended in-clinic manual therapy and exercises is available in Additional File III of Sullivan et al. [17] Exercise selection and progression in-clinic are guided by therapist expertise and clinical reasoning accounting for patient-specific variables including impairments and activity limitations. The TeMPO protocol calls for a total of 14 in-clinic PT sessions over a three-month treatment period. Study participants are asked to perform the TeMPO HEP in conjunction with the in-clinic PT so that a total of 100 min of strengthening exercises are performed per week.

#### TeMPO home exercise program

The TeMPO HEP consists of three primary components: 1) stretching; 2) strengthening; 3) functional activities. TeMPO recommends four 25-min exercise program sessions per week totaling 100 min of exercise. Specific exercises, directions for performance, rationale for progression, adjustments, and how to address pain are explained and easily accessible in a pamphlet and video [17]. All study participants are provided with an adjustable ankle weight in 1 pound increments up to 10 pounds. The home exercises are designed to progress exercises to maintain adequate dose for strengthening using these adjustable one-pound ankle cuff weights. Instructions for progression considered soreness and pain within 24 h of completing a HEP session to provide adequate rigor but remains easily understandable by study participants without therapist direction. The complete set of instructions given to TeMPO subjects have been published previously as Additional File I in Sullivan et al. [17]

#### Home exercise program modifications

To optimize the home exercise program the regimen includes multiple alternate forms of exercises for each major muscle group to allow someone experiencing difficulty completing a certain exercise to continue participating in the program. Instructions for the use of the incremental ankle weights were included so that the program could be modified to fit the different strength and fitness levels of the patients. To further make the program accessible to various styles of learning, an online and offline video (accessed via USB flash drive) and online and hardcopy pamphlet-based instructional materials were created. Specific guidelines are given about how to safely advance, hold or step back with progressions in addition to how to manage soreness and pain in the pamphlet.

## Discussion

The goals of this project were to develop both a standardized in-clinic PT intervention and HEP for persons with symptomatic degenerative meniscal tears in the setting of OA for the TeMPO RCT. We achieved our stated goals by incorporating evidence and input from a variety of sources, including existing literature, an expert panel of experienced clinicians, health care researchers and utilizing survey results from 21 licensed experienced therapists from across the US. We designed a comprehensive and easy to follow self-administered home exercise program that includes stretching, resisted strengthening exercises and guidelines for progression, adjustments and ways to manage soreness and pain. This exercise program requires minimal equipment and can be completed within a reasonable time frame (25 min). Our program specifies the major muscle groups for strengthening, the targeted muscles for stretching, and includes neuromuscular functional training.

Overall the PT intervention and home exercise program align with literature describing perioperative meniscectomy rehabilitation programs [9, 22–24, 29, 30] and international programs created to address degenerative meniscal tears or OA [6, 36, 37]. The TeMPO program is influenced by the PT regimen described in recent trials [24, 30, 38–41]. While these programs were all different, each of them focused primarily on strengthening.

One notable addition to the TeMPO program is that we incorporate gluteus medius exercises, which are more often strengthened in the context of hip pain, but have been demonstrated to play a pivotal role in rehabilitation for patients with knee pain [42]. It is unclear as to why other programs did not include this feature when it is considered part of routine care in physical therapy for patients with a variety of knee diagnoses. The gluteus medius is a primary stabilizer of the lower extremity in the frontal plane and works eccentrically to decelerate adduction and internal rotation of the hip leading to dynamic valgus at the knee, minimizing shear stress which is thought to cause meniscal injury. The TeMPO in-clinic program and HEP both incorporate strengthening exercises for the gluteus medius muscle.

Another feature of the TeMPO in-clinic PT program is the inclusion of manual therapy, which appears not to have been incorporated in PT regimens assessed previously in this patient population. Manual therapy has been shown to be effective in patients with a variety of knee-related diagnoses [15, 47] and was used by over 90% of therapists in our survey. The TeMPO in-clinic program and HEP both incorporate stretching to promote adequate range of motion and improve patients’ ability to perform stretching and strengthening exercises.

The TeMPO program was influenced by the work of Roos et al., Bennell et al. and the APTA clinical practice guidelines who outlined PT regimens with neuromuscular strengthening components [36, 37]. Compared to regimens recommended by our Scandinavian colleagues, the TeMPO in-clinic PT regimen was modified to require fewer hours of outpatient PT because time commitments among potential participants in the US, particularly among those who are employed, are often limited. Additionally, insurers in the US often limit the time of a PT session or number of PT sessions that can be reimbursed. We wanted to create a practical, effective, and pragmatic program for use within the US and thus realistic time management and billing issues were important considerations when creating the in-clinic program and HEP. This element of feasibility is emphasized in the MRC guidance [18].

This work is unique in that it is the first therapeutic program designed for the rehabilitation of symptomatic meniscal tear in the setting of OA that has been created and vetted by an international coalition of expert health science researchers, orthopaedic surgeons, rheumatologists and physical therapists. In addition, the TeMPO home exercise program is notable for providing a flexible yet standardized set of options that can be undertaken by a patient without input from a physical therapist. The inclusion of a weight progression and alternate exercises makes the HEP accessible across different physical ability levels and disease states while guidelines for excessive soreness and pain during or after exercise improve the safety of the HEP and the ability of patients to perform the exercises independently. The HEP is also unique in that it is accessible via mixed media sources including USB flash drives, online videos, and paper as compared to traditional paper handouts used as home therapy instructions. With the addition of digital components, the TeMPO HEP can be accessed from rural areas as well as densely populated cities.

We note that the interventions developed for the TeMPO trial are largely similar to those used for patients with knee OA in the absence of symptomatic meniscal tear. Our expert clinicians noted that the population of patients with both OA and MT may have more heterogenous symptom expression and impairments than those with OA alone (e.g. more swelling). Thus, the TeMPO interventions provide considerable flexibility of the regimen across and within patients.

This work has several limitations. For both the expert panel and PT survey, we used a convenience sample of collaborators and their national contacts respectively, which may have affected the viewpoints represented. We acknowledge the tension between personalization and standardization in designing interventions. Physical therapists typically provide individualized programs for patients and sessions can vary across time based on observed impairments and activity limitations. Both the TeMPO HEP and in-clinic PT intervention sacrifice some of this personalization. Here, we provided standardization of a home program and PT in-clinic intervention. The in-clinic PT program included variations for choices of interventions in the PT sessions and alternatives exercises for the HEP to accommodate various abilities.

## Final conclusions

This project describes the process of developing a standardized in-clinic PT program and HEP for the TeMPO RCT. The first is an in-clinic physical therapist directed manual therapy, stretching, strengthening and functional/neuromuscular exercise program that includes weights, machines, and flexible progression guided by physical therapist expertise. The second is a practical HEP that is performed independent of a physical therapist, requires minimal equipment and is comprised of three components: 1) stretching; 2) strengthening; and 3) functional activities. These regimens will form the components of the in-clinic PT intervention and HEP for the TeMPO trial, in which we hope to gain insight into the comparative effectiveness of these interventions and contextual effects for persons with symptomatic degenerative meniscal tear and OA.

## Supplementary information

**Additional file 1.** Email Survey of Physical Therapists.

## Data Availability

Not applicable.

## Abbreviations

ACSM: American College of Sports Medicine
APTA: American Physical Therapy Association
HEP: Home Exercise Program
OA: Osteoarthritis
PT: Physical Therapy
RCT: Randomized Controlled Trial
SD: Standard Deviation
TeMPO: Treatment of Meniscal Tear in Osteoarthritis (trial name)
US: United States

## References

[1] Lawrence Reva C., Felson David T., Helmick Charles G., Arnold Lesley M., Choi Hyon, Deyo Richard A., Gabriel Sherine, Hirsch Rosemarie, Hochberg Marc C., Hunder Gene G., Jordan Joanne M., Katz Jeffrey N., Kremers Hilal Maradit, Wolfe Frederick (2007). Estimates of the prevalence of arthritis and other rheumatic conditions in the United States: Part II. Arthritis & Rheumatism.

[2] Deshpande BR, Katz JN, Solomon DH, Yelin EH, Hunter DJ, Messier SP, Suter LG, Losina E (2016). Number of persons with symptomatic knee osteoarthritis in the US: impact of race and ethnicity, age, sex, and obesity. Arthritis Care Res (Hoboken).

[3] Bhattacharyya T, Gale D, Dewire P, Totterman S, Gale ME, McLaughlin S, Einhorn TA, Felson DT (2003). The clinical importance of meniscal tears demonstrated by magnetic resonance imaging in osteoarthritis of the knee. J Bone Joint Surg Am.

[4] Englund M, Guermazi A, Gale D, Hunter DJ, Aliabadi P, Clancy M, Felson DT (2008). Incidental meniscal findings on knee MRI in middle-aged and elderly persons. N Engl J Med.

[5] Lim HC, Bae JH, Wang JH, Seok CW, Kim MK (2010). Non-operative treatment of degenerative posterior root tear of the medial meniscus. Knee Surg Sports Traumatol Arthrosc.

[6] Stensrud S, Roos EM, Risberg MA (2012). A 12-week exercise therapy program in middle-aged patients with degenerative meniscus tears: a case series with 1-year follow-up. J Orthop Sports Phys Ther.

[7] Osteras H, Osteras B, Torstensen TA (2012). Medical exercise therapy, and not arthroscopic surgery, resulted in decreased depression and anxiety in patients with degenerative meniscus injury. J Bodyw Mov Ther.

[8] Hall M, Hinman RS, Wrigley TV, Roos EM, Hodges PW, Staples MP, Bennell KL (2015). Neuromuscular exercise post partial medial Meniscectomy: randomized controlled trial. Med Sci Sports Exerc.

[9] Dias JM, Mazuquin BF, Mostagi FQRC, Lima TB, Silva MAC, Resende BN, RMBd S, Lavado EL, Cardoso JR (2013). The effectiveness of postoperative physical therapy treatment in patients who have undergone arthroscopic partial Meniscectomy: systematic review with meta-analysis. J Orthop Sports Phys Ther.

[10] Ericsson YB, Dahlberg LE, Roos EM (2009). Effects of functional exercise training on performance and muscle strength after meniscectomy: a randomized trial. Scand J Med Sci Sports.

[11] St-Pierre DMM, Laforest S, Paradis S, Leroux M, Charron J, Racette D, Dalzell M (1992). Isokinetic rehabilitation after arthroscopic meniscectomy. Eur J Appl Physiol Occup Physiol.

[12] Matthews P, St-Pierre DM (1996). Recovery of muscle strength following arthroscopic meniscectomy. J Orthop Sports Phys Ther.

[13] Moffet H, Richards CL, Malouin F, Bravo G, Paradis G (1994). Early and intensive physiotherapy accelerates recovery postarthroscopic meniscectomy: results of a randomized controlled study. Arch Phys Med Rehabil.

[14] Vervest AM, Maurer CA, Schambergen TG, de Bie RA, Bulstra SK (1999). Effectiveness of physiotherapy after meniscectomy. Knee Surg Sports Traumatol Arthrosc.

[15] Goodwin PC, Morrissey MC, Omar RZ, Brown M, Southall K, McAuliffe TB (2003). Effectiveness of supervised physical therapy in the early period after arthroscopic partial meniscectomy. Phys Ther.

[16] Jokl P, Stull PA, Lynch JK, Vaughan V (1989). Independent home versus supervised rehabilitation following arthroscopic knee surgery--a prospective randomized trial. Arthroscopy.

[17] Sullivan JK, Irrgang JJ, Losina E, Safran-Norton C, Collins J, Shrestha S, Selzer F, Bennell K, Bisson L, Chen AT (2018). The TeMPO trial (treatment of meniscal tears in osteoarthritis): rationale and design features for a four arm randomized controlled clinical trial. BMC Musculoskelet Disord.

[18] Craig P, Dieppe P, Macintyre S, Michie S, Nazareth I, Petticrew M. Medical Research Council G: developing and evaluating complex interventions: the new Medical Research Council guidance. BMJ. 2008;337:–a1655. 10.1136/bmj.a1655 PMC2769032 18824488

[19] Bennell KL, Egerton T, Pua Y-H, Abbott JH, Sims K, Buchbinder R (2011). Building the rationale and structure for a complex physical therapy intervention within the context of a clinical trial: a multimodal individualized treatment for patients with hip osteoarthritis. Phys Ther.

[20] Hauk L (2014). Treatment of knee osteoarthritis: a clinical practice guideline from the AAOS. Am Fam Physician.

[21] Logerstedt DS, Scalzitti DA, Bennell KL, Hinman RS, Silvers-Granelli H, Ebert J, Hambly K, Carey JL, Snyder-Mackler L, Axe MJ (2018). Knee pain and mobility impairments: meniscal and articular cartilage lesions revision 2018. J Orthop Sports Phys Ther.

[22] Gauffin H, Tagesson S, Meunier A, Magnusson H, Kvist J (2014). Knee arthroscopic surgery is beneficial to middle-aged patients with meniscal symptoms: a prospective, randomised, single-blinded study. Osteoarthr Cartil.

[23] Herrlin SV, Wange PO, Lapidus G, Hallander M, Werner S, Weidenhielm L (2013). Is arthroscopic surgery beneficial in treating non-traumatic, degenerative medial meniscal tears? A five year follow-up. Knee Surg Sports Traumatol Arthrosc.

[24] Katz JN, Brophy RH, Chaisson CE, de Chaves L, Cole BJ, Dahm DL, Donnell-Fink LA, Guermazi A, Haas AK, Jones MH (2013). Surgery versus physical therapy for a meniscal tear and osteoarthritis. N Engl J Med.

[25] Kise NJ, Risberg MA, Stensrud S, Ranstam J, Engebretsen L, Roos EM (2016). Exercise therapy versus arthroscopic partial meniscectomy for degenerative meniscal tear in middle aged patients: randomised controlled trial with two year follow-up. The BMJ.

[26] Skou ST, Lind M, Hölmich P, Jensen HP, Jensen C, Afzal M, Jørgensen U, Thorlund JB (2017). Study protocol for a randomised controlled trial of meniscal surgery compared with exercise and patient education for treatment of meniscal tears in young adults. BMJ Open.

[27] Stensrud S, Risberg MA, Roos EM (2015). Effect of exercise therapy compared with arthroscopic surgery on knee muscle strength and functional performance in middle-aged patients with degenerative meniscus tears: a 3-mo follow-up of a randomized controlled trial. Am J Phys Med Rehabil.

[28] Osteras H (2014). A 12-week medical exercise therapy program leads to significant improvement in knee function after degenerative meniscectomy: a randomized controlled trial with one year follow-up. J Bodyw Mov Ther.

[29] Sihvonen R, Paavola M, Malmivaara A, Itälä A, Joukainen A, Nurmi H, Kalske J, Järvinen TLN (2013). Arthroscopic partial Meniscectomy versus sham surgery for a degenerative meniscal tear. N Engl J Med.

[30] Yim JH, Seon JK, Song EK, Choi JI, Kim MC, Lee KB, Seo HY (2013). A comparative study of meniscectomy and nonoperative treatment for degenerative horizontal tears of the medial meniscus. Am J Sports Med.

[31] Herrlin S, Hallander M, Wange P, Weidenhielm L, Werner S (2007). Arthroscopic or conservative treatment of degenerative medial meniscal tears: a prospective randomised trial. Knee Surg Sports Traumatol Arthrosc.

[32] Kise NJ, Risberg MA, Stensrud S, Ranstam J, Engebretsen L, Roos EM. Exercise therapy versus arthroscopic partial meniscectomy for degenerative meniscal tear in middle aged patients: randomised controlled trial with two year follow-up. BMJ. 2016;354. 10.1136/bmj.i3740 PMC4957588 27440192

[33] Jansen MJ, Viechtbauer W, Lenssen AF, Hendriks EJ, de Bie RA (2011). Strength training alone, exercise therapy alone, and exercise therapy with passive manual mobilisation each reduce pain and disability in people with knee osteoarthritis: a systematic review. J Physiother.

[34] Garber CE, Blissmer B, Deschenes MR, Franklin BA, Lamonte MJ, Lee IM, Nieman DC, Swain DP (2011). American College of Sports Medicine position stand. Quantity and quality of exercise for developing and maintaining cardiorespiratory, musculoskeletal, and neuromotor fitness in apparently healthy adults: guidance for prescribing exercise. Med Sci Sports Exerc.

[35] Borg DN, Costello JT, Bach AJ, Stewart IB (2017). Perceived exertion is as effective as the perceptual strain index in predicting physiological strain when wearing personal protective clothing. Physiol Behav.

[36] Skou ST, Roos EM (2017). Good life with osteoArthritis in Denmark (GLA:D™): evidence-based education and supervised neuromuscular exercise delivered by certified physiotherapists nationwide. BMC Musculoskelet Disord.

[37] Ageberg E, Nilsdotter A, Kosek E, Roos EM (2013). Effects of neuromuscular training (NEMEX-TJR) on patient-reported outcomes and physical function in severe primary hip or knee osteoarthritis: a controlled before-and-after study. BMC Musculoskelet Disord.

[38] Buchbinder R (2013). Meniscectomy in patients with knee osteoarthritis and a meniscal tear?. N Engl J Med.

[39] Herrlin SV, Wange PO, Lapidus G, Hallander M, Werner S, Weidenhielm L (2013). Is arthroscopic surgery beneficial in treating non-traumatic, degenerative medial meniscal tears? A five year follow-up. Knee Surg Sports Traumatol Arthrosc.

[40] Jevsevar DS (2013). Treatment of osteoarthritis of the knee: evidence-based guideline, 2nd edition. J Am Acad Orthop Surg.

[41] Moseley JB, O'Malley K, Petersen NJ, Menke TJ, Brody BA, Kuykendall DH, Hollingsworth JC, Ashton CM, Wray NP (2002). A controlled trial of arthroscopic surgery for osteoarthritis of the knee. N Engl J Med.

[42] Powers CM (2010). The influence of abnormal hip mechanics on knee injury: a biomechanical perspective. J Orthop Sports Phys Ther.

[43] Wan X, Wang W, Liu J, Tong T (2014). Estimating the sample mean and standard deviation from the sample size, median, range and/or interquartile range. BMC Med Res Methodol.

[44] Abbott JH, Chapple CM, Fitzgerald GK, Fritz JM, Childs JD, Harcombe H, Stout K (2015). The incremental effects of manual therapy or booster sessions in addition to exercise therapy for knee osteoarthritis: a randomized clinical trial. J Orthop Sports Phys Ther.

[45] Deyle GD, Allison SC, Matekel RL, Ryder MG, Stang JM, Gohdes DD, Hutton JP, Henderson NE, Garber MB (2005). Physical therapy treatment effectiveness for osteoarthritis of the knee: a randomized comparison of supervised clinical exercise and manual therapy procedures versus a home exercise program. Phys Ther.

[46] Moss P, Sluka K, Wright A (2007). The initial effects of knee joint mobilization on osteoarthritic hyperalgesia. Man Ther.

[47] Perlman AI, Sabina A, Williams AL, Njike VY, Katz DL (2006). Massage therapy for osteoarthritis of the knee: a randomized controlled trial. Arch Intern Med.

[48] Weng MC, Lee CL, Chen CH, Hsu JJ, Lee WD, Huang MH, Chen TW (2009). Effects of different stretching techniques on the outcomes of isokinetic exercise in patients with knee osteoarthritis. Kaohsiung J Med Sci.

