# Supplementary material for: A consensus-based process identifying physical therapy and exercise treatments for patients with degenerative meniscal tears and knee OA: the TeMPO physical therapy interventions and home exercise program
Source: BMC Musculoskelet Disord. 2019 Nov 4;20:514. doi: 10.1186/s12891-019-2872-x (PMC6830005; doi:10.1186/s12891-019-2872-x)
Supplement: Supplementary file 1 — Additional file 1. Email Survey of Physical Therapists. [file 12891_2019_2872_MOESM1_ESM.docx]

**Additional file 1: Email Survey of Physical Therapists**

**TeMPO Trial**

**We are interested in how you treat patients in the 50-70 yo age range with degenerative meniscal tear. Imagine that you have evaluated such a patient and are providing him or her care.  On a typical follow-up treatment visit....**

1. How long would you usually spend with the patient?  __________ minutes

2. Do you administer supervised exercises?  _____ yes   _____ no

3. How long do you spend on supervised exercises? __________ minutes

4. Do you administer manual therapy?  _____ yes   _____ no

5. How long do you spend on manual therapy? __________ minutes

6. What types of manual therapy do you do?

i. Manual joint mobilization  ___ yes  ___ no

ii. Manual muscle stretching  ___ yes  ___ no

iii. Manual soft tissue mobilization   ___ yes   ___ no

7. Besides supervised exercises and manual therapy, what other treatments do you typically administer to these patients?

**A few other pieces of information would be useful for us:**

8. What is your work setting (academic medical center, private practice, etc.)?

9. In which city/state/country do you work?

10. How long have you been practicing independently as a physical therapist?

**Thank you!**
